# Incidents related to intrahospital transport of patients in the ICU

**DOI:** 10.1186/cc9955

**Published:** 2011-03-11

**Authors:** C Van Velzen, AH Brunsveld-Reinders, MS Arbous

**Affiliations:** 1 Leiden University Medical Center, Leiden, the Netherlands

## Introduction

The objective of this study was to determine the incidence and type of incidents related to intrahospital transport (IHT) of critically ill patients in our ICU and to identify contributing factors of these incidents.

## Methods

Since 2006 an electronic incident registration system was implemented on our tertiary university mixed adult ICU. Two investigators identified incidents related to IHT between 2006 and 2009. IHT incidents were categorized according to phase of occurrence: before, during or after IHT. The physical derangement of the patients could be cardiovascular, respiratory, or neurologic. By means of a structured incident analysis method, potential causal and contributory factors were determined.

## Results

In a 1-year period (2009) 568 transports were performed in 1,821 ICU patients. Thus about one-third of all ICU patients needed IHT. Of all incidents reported from 2006 to 2009, 2.1% was IHT related. IHT had an incident rate of 3.7%. Of all IHT incidents (*n *= 124), 35% occurred pre-transport, 50% during transport and 15% post-transport. Thirty-two patients (25.8%) had a physical derangement. The most involved organ system was the respiratory system (*n *= 20) followed by the cardiovascular system (*n *= 10) and neurological problems (*n *= 2). Indentified causes were technical (*n *= 39), organizational (*n *= 50) and human error (*n *= 86); more than one cause per case could be assigned. Technical causes occurred mostly during transport (*n *= 28) followed by pre-transport (*n *= 9) and after transport (*n *= 2). Contributing factors were mostly equipment related (*n *= 22). Human error was divided between during transport (*n *= 35) and pre-transport (*n *= 33) and fewer occurred after transport (*n *= 18). Contributing factors were coordination errors (*n *= 37) and external factors (*n *= 19). Organizational causes were mostly pre-transport (*n *= 26) and during transport (*n *= 20) and fewer after transport (*n *= 4). Almost all contributing factors were due to information transfer (*n *= 31). Overall, the most important contributing factors were coordination errors (*n *= 37), information transfer (*n *= 31), equipment failure (*n *= 22), and insufficient supervision (*n *= 22).

## Conclusions

Incidents related to IHT have an incidence of 3.7%. Most incidents occurred pre-transport and during transport. The incidents are predominantly on the respiratory and cardiovascular systems. Human failure is an important cause of IHT. Contributing factors were coordination errors, equipment failure, information transfer and insufficient supervision. Given the contributing factors we think the number of incidents could be reduced by means of a transportation checklist.

